# The *CaALAD* Gene From Pepper (*Capsicum annuum* L.) Confers Chilling Stress Tolerance in Transgenic *Arabidopsis* Plants

**DOI:** 10.3389/fpls.2022.884990

**Published:** 2022-04-29

**Authors:** Huiping Wang, Zeci Liu, Jianming Xie, Jing Li, Jing Zhang, Jihua Yu, Linli Hu, Guobin Zhang

**Affiliations:** College of Horticulture, Gansu Agriculture University, Lanzhou, China

**Keywords:** 5-aminolevulinic acid, hydrogen sulfide, reactive oxygen species, low temperature, cold tolerance

## Abstract

The *ALAD* gene encodes an enzyme that is essential for chlorophyll biosynthesis and is involved in many other physiological processes in plants. In this study, the *CaALAD* gene was cloned from pepper and sequenced. Multiple sequence alignment and phylogenetic analysis of ALAD proteins from nine plant species showed that *ALAD* is highly conserved, and that CaALAD shows the highest homology with the ALAD protein from eggplant. Subcellular localization indicated that the CaALAD protein is mainly localized to the chloroplasts. After transferring *CaALAD* into the *Arabidopsis thaliana* genome, cold tolerance of the transgenic lines improved. Overexpression of *CaALAD* increased the relative transcription of the *AtCBF2*, *AtICE1*, and *AtCOR15b* genes in transgenic *Arabidopsis* plants exposed to low temperature (4°C) stress, and the contents of reactive oxygen species decreased due to increased activities of superoxide dismutase, peroxidase, and catalase. Moreover, chlorophyll biosynthesis, as determined by the contents of porphobilinogen, protoporphyrin IX, Mg-protoporphyrin IX, prochlorophyllate, and chlorophyll in the transgenic *Arabidopsis* plants, increased in response to low temperature stress. In addition, the transgenic lines were more sensitive to exogenous ALA and NaHS, and the H_2_S content of transgenic line plants increased more rapidly than in the wild-type, suggesting that *CaALAD* may respond to low temperatures by influencing the content of H_2_S, a signaling molecule. Our study gives a preliminary indication of the function of *CaALAD* and will provide a theoretical basis for future molecular breeding of cold tolerance in pepper.

## Introduction

Pepper is an important vegetable crop and condiment, and the normal growth temperature of pepper plants is 20–30°C (Guo, 2013). Growth and development are hindered if the temperature is below 15°C, and plants will be seriously damaged or can die if the temperature falls below 5°C (Wang et al., 2021a). In the winter and spring in northern China, low temperatures are the main abiotic stress that limits the growth, development, and yield of pepper (Tang et al., 2021). Low temperatures will reduce stomatal conductance, change the biological characteristics of thylakoid membranes, reduce chlorophyll content, and limit photosynthetic electron transfer, thus reducing the photosynthetic capacity of plants (Allen and Ort, 2001; Tang et al., 2021). Low temperatures can also lead to the production of reactive oxygen species (ROS), which cause oxidative damage to plants (Wang et al., 2021b).

In order to resist the effects of low temperature, plants have evolved complex response mechanisms to low temperature stress, and cold-resistance genes play a key role. Cold tolerance in plants is regulated by multiple genes, and the low-temperature response transcription network composed of ICE1-CBF-COR is the most widely studied pathway in the plant response to cold. Previous studies have shown that expression of three members of the *CBF* gene family, *CBF1*, *CBF2*, and *CBF3*, can be induced briefly and rapidly by low temperature stress (Medina et al., 1999; Gilmour et al., 2010). The induced CBF transcription factor proteins can bind to the *cis*-elements present in the *COR* gene promoter and activate *COR* gene expression. ICE is a transcriptional activator that induces the expression of the *CBF* gene family at low temperatures. ICE can specifically bind to the promoter sequence of *CBF* at low temperatures to induce the expression of *CBF*, and the induced CBF protein then binds to the DRE sequence in the promoter of its downstream target gene to induce the expression of *COR*, thus improving frost tolerance in plants (Tang et al., 2020).

Our previous studies showed that 5-aminolevulinic acid (ALA) and hydrogen sulfide enhanced the photosynthesis and antioxidant capacity of cold-sensitive pepper plants (Wang et al., 2021b). We found that ALA and H_2_S significantly induced the expression of *ALAD* in response to chilling stress (Wang et al., 2021b). Therefore, we hypothesized that *ALAD* might be involved in regulating the resistance to low temperature stress in pepper.

The non-proteinogenic amino acid ALA is a common precursor in the biosynthesis of tetrapyrroles in plants, and ALA is considered to be a new plant growth regulator (Wu et al., 2018b). ALA can regulate plant growth and development, vegetative growth, seed germination, and fruit coloring (Zhang, 2010; Zhao et al., 2013; Kobayashi and Masuda, 2016; Wu et al., 2018a,b). In addition, ALA regulates certain metabolic processes, such as chlorophyll, heme, and siroheme biosynthesis, and alleviates abiotic stress by regulating photosynthesis, nutrient absorption, antioxidant defense, and osmotic regulation (Kim et al., 2014; Wu et al., 2018b). 5-aminolevulinate dehydratase (ALAD) is a necessary enzyme for the biosynthesis of tetrapyrroles, and it catalyzes the condensation of two molecules of ALA to form porphobilinogen (PBG; Yang, 2008). Therefore, the activity of ALAD directly determines the metabolism and accumulation of ALA in plants (Long et al., 2013). Moreover, ALA does not generally accumulate in developing leaves, but is directly transformed into PBG by ALAD (Killiny et al., 2018). At present, the *ALAD* gene has been successfully cloned from spinach (Berglund and Tegenfeldt, 1992), soybean (Boese et al., 1991), tomato (Polking et al., 1995), pea (Cheung et al., 1997), and some other plant species. Previous studies have shown that the *ALAD* gene sequence and structure are highly conserved (Yang, 2008; Lu, 2013).

The activity of ALAD is affected by temperature, pH, sugars, and mercaptan (Yang, 2008; Xu, 2014). D-fructose and D-glucose inhibit ALAD activity (Lee et al., 2003; Yang, 2008). Low concentrations of dithiothreitol can improve ALAD activity, but high concentrations can inhibit activity (Xu, 2014). Osmotic stress induced by sorbitol was found to inhibit ALAD activity in maize leaves (Jain et al., 2018). Also, ALAD activity strongly depends on the binding of metal ions (including Zn^2+^, Mn^2+^, and Mg^2+^). *In vitro* assays of ALAD enzyme activity found that Ca^2+^, Mn^2+^, and K^+^ significantly increase activity, while Cu^2+^ significantly inhibits the activity of ALAD (Long et al., 2013). In cucumber, it was found that Al^3+^ can significantly inhibit ALAD activity and growth in cucumber. Al^3+^ may affect the growth and development of plants by forming complexes with nucleotides, cell walls, and other biomolecules (Pereira et al., 2006).

Some previous studies have shown that the ALAD protein plays important roles in plant growth, development, and stress resistance. Lu (2013) found that in garlic, changes in ALAD activity were consistent with the PBG content and the degree to which the plants turn green through ALAD activity, and also the relative expression of *ALAD* in different varieties of garlic. The expression level of *ALAD* was found to be much higher at low temperature than at normal temperature (Lu, 2013). Chai et al. (2017) found that the cotton lesion mimic mutant *Ghllm*, which results from a mutation in a gene encoding ALAD, causes necrotic spots on cotton leaves (Chai et al., 2017). This mutation leads to the accumulation of ALA, which in turn leads to the production of ROS, and thus induces the expression of *GhEDS1*, *GhPAL*, and *GhPAD4*, leading to an increase in the salicylic acid content (Chai et al., 2017). Also, the expression of SA leads to the increased expression of the *PR* gene, thus increasing the resistance of cotton to verticillium wilt (Chai et al., 2017). *ALAD1* overexpression in wheat improved ALA tolerance in tobacco plants (Yu et al., 2011). In addition, silencing of the *ALAD* gene can lead to plant stress, resulting in yellow necrotic spots on citrus leaves and stems, decreased levels of chlorophyll, starch, sucrose, *trans*-violet and *cis*-violet xanthin, α and β cryptoxanthin, and increased levels of zeaxanthin (Killiny et al., 2018). Studies in *Arabidopsis* using RNAi gene silencing and *HEMB1* (*ALAD*) mutants showed that the loss or reduction of *HEMB1* can seriously affect plant growth and development, resulting in seedling and embryo death (Tang et al., 2012). Wittmann et al. (2018) found that silencing the *TRX-M1*/*M2*/*M4* thioredoxin (TRX) genes leads to decreased stability of ALAD, and that TRX in chloroplasts can interact with ALAD to improve its activity in *Arabidopsis* (Wittmann et al., 2018). FHY3/FAR1 binds with the promoter of *ALAD* to initiate the expression of *ALAD* and enhance its activity, thus affecting the biosynthesis of chlorophyll (Tang et al., 2012). The results of our previous study also showed that the transcription of *ALAD (HEMB)* was induced by treatment with exogenous ALA and hydrogen sulfide in pepper seedlings under low temperature stress (Wang et al., 2021b). Therefore, it is necessary to understand the molecular mechanisms that underly cold tolerance mediated by ALAD in pepper.

In this study, the *CaALAD* gene was cloned from pepper and expressed in *Arabidopsis* to study its effect on low temperature stress tolerance. The relative cold tolerance of transgenic *Arabidopsis* plants overexpressing the pepper *ALAD* gene was determined by phenotypic and physiological indicators, and the results will establish a genetic foundation for the study of cold tolerance in pepper.

## Materials and Methods

### Plant Materials and Growth Conditions

Wild type (WT) Col-0 and *CaALAD*-overexpressing *Arabidopsis* lines, the pepper cultivar “Hangjiao 2” (Purchased from Shenzhou Lvpeng Company, Tianshui, China) and *Nicotiana benthamiana* were used in this experiment. *Arabidopsis* seeds were sown on substrate (a 1:1 mixture of grass charcoal soil and vermiculite) or sterile Murashige and Skoog (MS) medium and grown at 23°C and 65% relative humidity with a 16 h/8 h (light/dark) photoperiod and a light intensity of 200 μmol m^–2^ s^–1^ (RT). The pepper seeds were spread on a wet towel and incubated in the dark for 3–5 day at a constant temperature of 28°C. After the seeds had germinated, they were planted in a substrate consisting of a 1:3 mixture of vermiculite and cultivation substrate. *N. benthamiana* seedlings were grown on the same mixed substrate as pepper under the following conditions: relative humidity 65%; temperature and photoperiod = 28°C/18°C (12 h light/12 h dark); and light intensity = 300 μmol m^–2^ s^–1^.

*Arabidopsis* seeds are rigorously sterilized before sowing on MS medium. WT and transgenic *Arabidopsis* seeds were surface sterilized with 75% alcohol for 1 min, washed with sterile water once and then treated with 1% sodium hypochlorite for 10 min, washed with sterile water five times, and cultured on MS medium with or without 50 mg/L kanamycin antibiotic. Kanamycin was used to screen genetically modified seeds for the presence of the Km*^r^* gene that is linked to the *CaALAD* transgene, and the third generation of the transgenic lines (T_3_) that were screened were used in the cold tolerance tests.

The seeds of WT and transgenic *Arabidopsis* were grown on MS medium for 1 week and transferred to square petri dishes. The seeds were cultured vertically at 4°C for another week and the effects of low temperature on the growth of the plants were then observed. The 25-day-old WT and transgenic seedlings which were sown on substrate were transferred to 4°C (LT) for 7 days. Samples were taken at different times during this period to determine the various indicators of cold stress or tolerance. Seedlings grown under regular conditions (23°C, RT) were used as the controls.

### Cloning of the *CaALAD* Gene

To clone *CaALAD* from *C. annuum*, the *ALAD* gene sequence of *Arabidopsis* was downloaded from the TAIR database^1^, this sequence was used as a query in BLAST searches of the pepper database; the gene with the highest sequence homology to *Arabidopsis ALAD* was named *CaALAD*. A pair of gene-specific primers, TL-F and TL-R, were designed using Primer 5 based on the *CaALAD* sequence (Table 1). The full-length cDNA of *CaALAD* was obtained *via* PCR amplification using *C. annuum* total RNA, the gene-specific primer pair, and Tks Gflex DNA Polymerase (TaKaRa, Japan). The PCR products were then cloned into the pEASY^^®^^ -Blunt Simple Cloning Vector (TransGene, Beijing, China) and transferred into *Escherichia coli* strain Trans1-T1 (Transgene) for DNA sequencing.

**TABLE 1:** Primers used for PCR.

Gene name	Primer *F* sequence (5′-3′)	Primer R sequence (5′-3′)
ALAD	ATGGCTTCCACGGCAATG	TTATGATCCGGCTAGCTTATACC
pYBA1132-ALAD	gcggtggcggccgctATGGCTTCCACGGCAATG	ACCGGGCCCCCCCTCGAGTGATCCGGCTAGCTTATACCC
pBI121-ALAD	ttggagagaacacgggggactATGGCTTCCACGGCAATG	AGGGACTGACCACCCGGGGATCTTATGATCCGGCTAGCTTATACC

### *Arabidopsis* Transformation and Selection of Transgenic Plants

The amplified target DNA fragment was cloned into the pBI121 vector by homologous recombination, and the restriction sites on the vector were Xbal and BamH1. Recombinant pBI121-*CaALAD* plasmids were introduced into the *Agrobacterium* strain GV3101. *Agrobacterium*-mediated transformation was performed to produce T_0_-generation transgenic lines using the floral dip method (Martins et al., 2015). Transgenic *Arabidopsis* T_1_-generation seeds were screened on solid MS medium containing 50 mg/L kanamycin. Homozygous T_3_-generation lines were used in the experiments.

### Sequence Alignment and Evolutionary Analyses of ALAD Proteins From Different Plant Species

The homologous *ALAD* genes of tomato (*Solanum lycopersicum*), potato (*Solanum tuberosum*), eggplant (*Solanum melongena*), tobacco (*Nicotiana tabacum*), *Arabidopsis thaliana*, rice (*Oryza sativa*), wheat (*Triticum aestivum*), and maize (*Zea may*) were obtained by BLAST searches of the NCBI database using the deduced protein sequence of *CaALAD* as the query. The phylogenetic relationships among the predicted ALAD proteins of these nine species were analyzed using DNAMAN 6.0 (Lynnon Biosoft, Montreal, Canada). A phylogenetic analyses was performed using the neighbor-joining method as implemented in MEGA 6.0 with 1,000 bootstrap replicates (Taghizadeh et al., 2020). The functional domains and tertiary structure of CaALAD were predicted using the conserved domain database (CDD)^2^, and SWISS-MODEL^3^.

### Subcellular Localization of *CaALAD*

To determine the subcellular localization of *CaALAD*, we cloned *CaALAD* (without the termination codon) into the pYBA1132 vector to give an in-frame fusion with the EGFP reporter protein gene. As with the pBI121 vector, the restriction sites on the pYBA1132 vector were also Xbal and BamH1. The p35S:CaALAD-EGFP and 35S:EGFP control plasmids were transformed into *Agrobacterium* GV3101, and the recombinant strains were infiltrated into 6–8 tobacco (*Nicotiana tabacum*) leaves with a syringe as described previously (Sparkes et al., 2006). The EGFP signals in leaf epidermal cells were subsequently observed under a fluorescence microscope (Zeiss Vert.A1, Oberkochen, Germany).

### RNA Isolation and Real-Time Quantitative PCR Analysis

Total RNA was extracted from plant tissues using an RNA Extraction Kit (Tiangen, Beijing, China) according to the manufacturer’s instructions. First-strand cDNA was synthesized from total RNA using EasyScript^®^ One-Step gDNA Removal and cDNA Synthesis SuperMix (Transgene, Beijing, China). The *AtUBQ4* gene was used as the reference for normalization of gene expression. The primer sequences used for amplification of *AtUBQ4*, *CaALAD*, and other cold-tolerance genes (*AtCBF1, AtCBF2, AtCOR15a, AtCOR15b, AtICE1*, and *AtICE2*) are given in Table 2 (Wang et al., 2021b). All oligonucleotide primers were synthesized by Shenggong (Shanghai, China).

**TABLE 2:** Oligonucleotide primers used for qRT-PCR assays in this study.

Gene name	Primer *F* sequence (5′-3′)	Primer *R* sequence (5′-3′)
*AtUBQ4*	GGGCACTCAAGTATCTTGTTAGC	TGCTGCCCAACATCAGGTT
*AtICE1*	GTTCGGGAATGAGGAGGTTTAG	AACACTCTCAGCCGCTTTAC
*AtICE2*	TCCACAAACGCTGTCTTACC	GTTCACTGCCTTTCCTTCTCT
*AtCBF1*	GCATGTCTCAACTTCGCTGA	ATCGTCTCCTCCATGTCCAG
*AtCBF2*	GTTTCCTCAGGCGGTGATTA	TCAACTCACACACCCACTTAC
*AtCOR15a*	GGCCACAAAGAAAGCTTCAG	CTTGTTTGCGGCTTCTTTTC
*AtCOR15b*	CTCAACGAAGCCACAAAGAAAG	CTTCCTCAGTCGCAGTTTCA
*CaALAD*	GCAGTAAGAGCTGCATTCCAAG	TTGAACAAGACCATGCCTCCA

### Semi-Quantitative PCR

To quantify the expression of *CaALAD* in transgenic *Arabidopsis* plants overexpressing the pepper *ALAD* gene, RNA extracted from the transgenic and wild-type (WT) plants was amplified by PCR using RT-PCR primers specific for *CaALAD* (*HEMB*) and reference primers for the *Arabidopsis* gene *AtUBQ4*, followed by agarose gel electrophoresis. The annealing temperature was set to 58°C, and 32 cycles of amplification were used.

### Determination of Protoporphyrin IX, Mg-Protoporphyrin IX, Protochlorophyllide, and Chlorophyll Contents

The contents of Protoporphyrin IX (Proto IX), Mg-protoporphyrin IX (Mg-proto IX), Protochlorophyllide (Pchl), and Chl in WT and transgenic plants were determined at 0 days and 7 days after low temperature (4°C) treatment. The methods used are the same as those used in our previous study (Wang et al., 2021b).

### Determination of Porphobilinogen Content

The PBG contents of WT and transgenic plants were determined at 0, 1, and 7 days after low temperature treatment. The determination of PBG content was based on the method of Kayser et al. (2005) with slight modifications. The details are as follows: 1.0 *g* of leaf blade was ground in an ice bath with 4 mL of 0.05M Tris–HCl (pH = 8.0), and vortex mixed. The slurry was centrifuged at 12,000 rpm at 4°C for 10 min, and the supernatant was absorbed into a new EP tube. One milliliter of ALA buffer (0.15 *g* ALA dissolved in 50 mL 0.05 mol/L Tris–HCl, pH 8.0) was added, and the mixture was mixed well. One volume of Ehrlich’s reagent (84 mL glacial acetic acid, 16 mL 70% perchloric acid, 2 *g* dimethylaminobenzaldehyde) was added and the solution was incubated in the dark for 15 min. The tube was then centrifuged at 12,000 rpm for 10 min, and the absorbance was then measured at 555 nm. The concentration was calculated according to the molar extinction coefficient of PBG (6.1 × 10^4^ mol^–1^ cm^–1^). PBG concentration = A_552_/(molar extinction coefficient × diameter).

### Histochemical Staining and Determination of Hydrogen Peroxide (H_2_O_2_) and Superoxide Anion (O_2_^•–^) Contents, and the Activities of Antioxidant Enzymes

The H_2_O_2_ and O_2_^•–^ contents were determined by the nitroblue tetrazolium [NBT, 0.1% (w/v)] and 3,3-diaminobenzidine (DAB, 1 mg/ml) uptake methods, respectively, as previously described (Xianyang et al., 2011; Ma et al., 2018). The contents of H_2_O_2_ and superoxide anion O_2_^•–^, as well as the activities of the antioxidant enzymes superoxide dismutase (SOD), peroxidase (POD), and catalase (CAT) were determined before and after 1 day of low temperature stress. These indicators were measured using kits purchased from Comin Biotechnology (Suzhou, China) following the manufacturer’s instructions.

### Seed Germination Test

T_3_-generation transgenic and WT seeds were soaked in solutions containing 100 μmol/L NaHS, 500 μmol/L NaHS, and 1,000 μmol/L NaHS for 12 h. The transgenic and WT seeds were soaked in 1 mg/L ALA, 10 mg/L ALA, and 100 mg/L ALA for 24 h, then washed once with sterile water. The seeds were sterilized with 75% alcohol for 1 min, washed once with sterile water, and sterilized with 1% sodium hypochlorite for 10 min. After washing with sterile water five times, the seeds were sown on MS medium and the germination rate was calculated after 5 days.

### Determination of H_2_S Content

The H_2_S contents of the WT and transgenic plants were determined after 0, 2, 6, 12, and 24 h at 4°C using a kit obtained from Comin Biotechnology (Suzhou, China).

### Statistical Analysis

All experiments consisted of three independent replicates, and SPSS 22.0 (SPSS Institute Inc., United States) was used to determine statistical significance at *p < 0.05 and ^**^p < 0.01; Student’s *t*-test was used to analyze the significance difference of the means. Origin 9 software (OriginLab Institute Inc., United States) was used for drawing the figures.

## Results

### *CaALAD* Sequence and Phylogenetic Analysis

**FIGURE 1:**
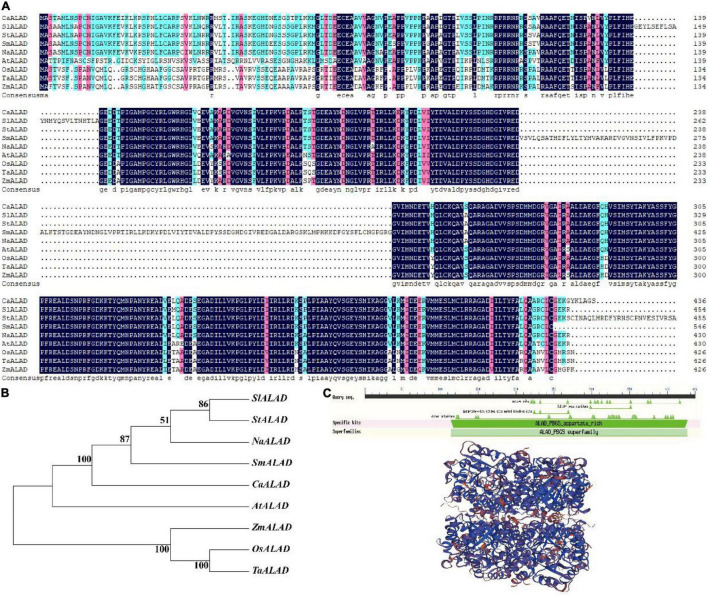
Sequence alignment and phylogenetic relationship between *CaALAD* and its homologous genes from other species. **(A)** *ALAD* protein sequences alignment of the pepper (*CaALAD*), tomato (*SlALAD*), potato (*StALAD*), eggplant (*SmALAD*), tobacco (*NaALAD*), *Arabidopsis* (*AtALAD*), rice (*OsALAD*), wheat (*TaALAD*), and maize (*ZmALAD*). Identical residues are displayed by mazarine. **(B)** Phylogenetic tree of *CaALAD* and its homologous proteins from tomato, potato, eggplant, tobacco, *Arabidopsis*, rice, wheat, and maize. **(C)** The predicted *CaALAD* functional domain and tertiary structure.

A phylogenetic tree derived from a multiple protein sequence alignment showed that the ALAD proteins are highly homologous in the nine plant species examined (Figure 1A). As shown in Figure 1B, the pepper ALAD protein is most closely related to the protein from eggplant; the ALAD proteins from five species in the *Solanaceae* plus *Arabidopsis* form a well-supported clade, while the proteins from three monocot species form a second well-supported clade, which also indicates that ALAD is a highly evolutionarily conserved protein in higher plants. The domain prediction results show that the CaALAD contains a single ALAD domain, which implies that CaALAD belongs to the ALAD family (Figure 1C). As with the domain prediction result, the predicted tertiary structure also shows that the CaALAD protein consists of one main part, and there are many folding and crimping in the protein (Figure 1C).

### Subcellular Localization of *CaALAD* in *Nicotiana benthamiana* and the Expression of *CaALAD* in *Arabidopsis*

**FIGURE 2:**
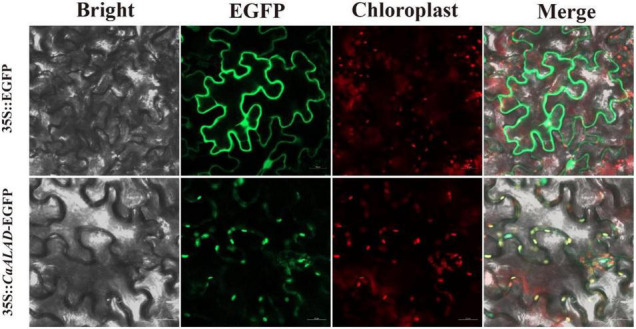
Subcellular localization of CaALAD in *N. benthamiana* leaf cells. Fluorescence microscopy of the 35S:*CaALAD*-EGFP and 35S:EGFP (control) plasmids. Scale bars = 20 μm.

The subcellular localization analysis showed that the control EGFP fluorescence signal was evenly distributed throughout the *N. benthamiana* leaf epidermal cells, while the signal from the *CaALAD*-EGFP fusion protein was localized to the chloroplasts (Figure 2).

### Effects of Overexpression of *CaALAD* on Growth of Transgenic *Arabidopsis* Plants Under Low Temperature Stress

**FIGURE 3:**
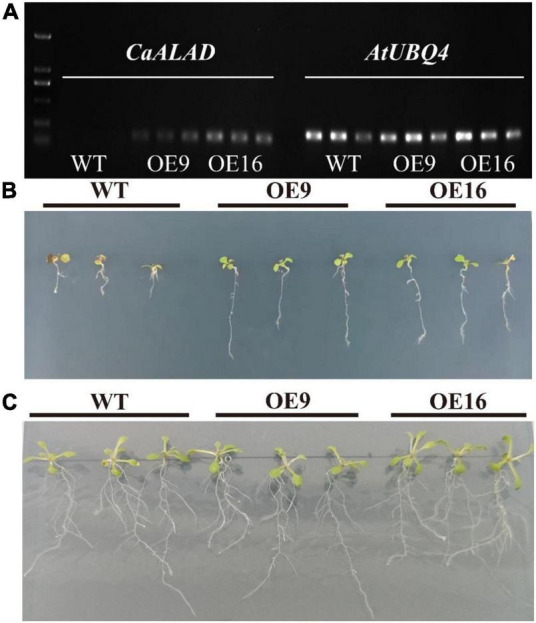
Effects of the overexpression of *CaALAD* on growth in transgenic *Arabidopsis* Col-0 plants exposed to low temperature stress for 1 week. WT: wild-type *Arabidopsis thaliana* Col-0. OE: the *CaALAD*-overexpressing (OE) lines. **(A)** Electrophoresis results of *CaALAD* in *A. thaliana* by semi-quantitative PCR. **(B)** The phenotypes of WT and OE plants that were exposed to low temperature stress at 4°C for 1 week. **(C)** The phenotypes of WT and OE plants grown at 23°C for 1 week.

In order to examine the expression of *CaALAD* in *Arabidopsis*, the reference gene *AtUBQ4* and the *CaALAD*-specific primers were used to conduct semi-quantitative PCR using cDNA of the WT and *CaALAD*-overexpressing transgenic plants as the templates. The results are shown in Figure 3A; there is no band from the amplification of WT *Arabidopsis* cDNA with the primer pair specific for *CaALAD*. The bands amplified with the *AtUBQ4* primers were clear, and the *CaALAD*-overexpressing lines had clear bands amplified with the *CaALAD*-specific primers. This indicates that the *CaALAD* gene is successfully transcribed in the transgenic *Arabidopsis* plants.

The growth of transgenic plants after 1 week of exposure to low temperature stress compared to normal culture conditions is shown in Figures 3B,C. However, the growth of *CaALAD*-overexpressing transgenic plants was significantly better than that of WT plants after low temperature stress, and the transgenic plants were less damaged by exposure to low temperature than were the WT plants.

### Overexpression of *CaALAD* Increased the Transcription of Cold Tolerance Genes in Transgenic *Arabidopsis*

**FIGURE 4:**
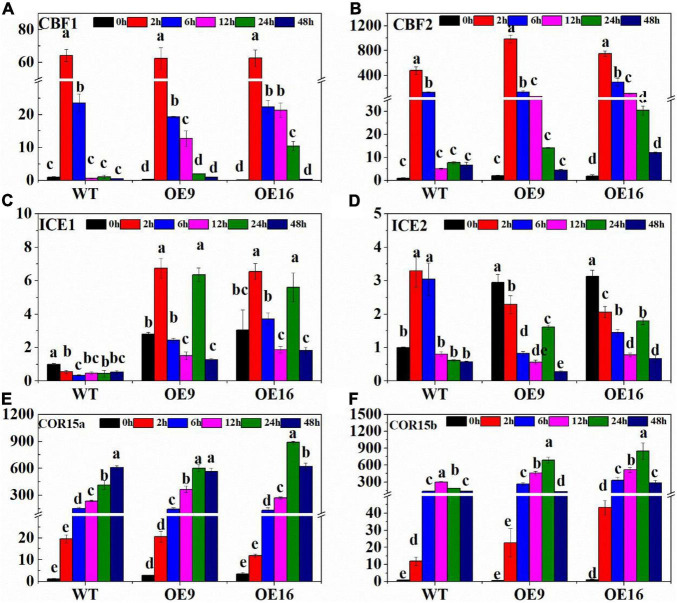
The relative expression of cold stress-related genes including *AtCBF1* **(A)**, *AtCBF2* **(B)**, *AtICE1* **(C)**, *AtICE2* **(D)**, *AtCOR15a* **(E)**, and *AtCOR15b* **(F)** in wild-type (WT) and transgenic plants exposed to low temperature stress for 0, 2, 6, 12, and 24 h. OE: the *CaALAD*-overexpressing (OE) lines. The error bars represent the standard errors for three independent tests. Different letters indicate significant differences (*p* < 0.05) based on Duncan’s test.

In order to identify the effect of *CaALAD* on cold tolerance in transgenic *Arabidopsis*, the relative expression of six cold stress-related genes, *AtCBF1*, *AtCBF2*, *AtICE1*, *AtICE2*, *AtCOR15a*, and *AtCOR15b*, was measured at different times during low temperature stress (Figure 4). In response to low temperature stress, the transcription of *AtCBF1* in both WT and the *CaALAD* transgenic lines increased sharply at first, then decreased, and reached its highest expression level at 2 h (Figure 4A). At 0 h, the expression level of *AtCBF1* in plants of the two transgenic lines was only 25% of that in WT plants (Figure 4A). *AtCBF1* expression in the WT decreased rapidly by 97.3% at 12 h under low temperature stress compared with 6 h, but it decreased by 34.1 and 4.8% in OE9 and OE16, respectively, (Figure 4A). The expression profiles of *AtCBF2* were similar to those of *AtCBF1*. The relative expression of *AtCBF2* also increased rapidly at 2 h and then gradually decreased from 6 to 48 h (Figure 4B). The initial expression of *AtCBF2* in the transgenic plants at 0 h was the 2-fold higher than in WT (Figure 4B). At 2 h, the expression of *AtCBF2* in the OE9 and OE16 lines increased by 1.1- and 1.6-fold, respectively, compared with WT (Figure 4B). At 12 h, the expression of *AtCBF2* in WT decreased drastically by 96% compared with 6 h, while the expression of *AtCBF2* in OE9 and OE16 decreased by only 53.8 and 63%, respectively, (Figure 4B). After low temperature stress, the expression of *AtICE1* in WT decreased, while the expression of *AtICE1* in the plants of both transgenic lines increased at 2 h, then decreased, and then increased at 24 h (Figure 4C). At 2 h, the expression of *AtICE1* in OE9 and OE16 plants increased 11.2- and 10.9-fold, respectively, compared with WT (Figure 4C). During low temperature stress, the relative expression of *AtICE2* in the WT increased first and decreased significantly after 6 h (Figure 4D). However, the relative expression of *AtICE2* in the transgenic plants showed a more gradual decrease from 6 to 12 h of low temperature stress, then increased by 2- to 3-fold at 24 h, before decreasing again at 48 h (Figure 4D). At 0 h, the relative expression of *AtICE2* in OE9 and OE16 plants increased 1.9- and 2.1-fold, respectively, compared with WT (Figure 4D). The relative expression of *AtCOR15a* in both WT and transgenic plants increased rapidly, beginning at 2 h of low temperature stress, but the expression in the transgenic plants decreased at 48 h while expression in the WT peaked at 48 h (Figure 4E). In response to low temperature stress treatment, the relative expression of *AtCOR15b* in WT and transgenic plants showed similar profiles in that first expression increased and then decreased (Figure 4F). The expression level of *AtCOR15b* in the WT peaked at 6 h, while it peaked in the OE lines at 12 h. Relative expression of *AtCOR15b* was significantly higher in the transgenic plants at 2, 6, 12, and 24 h than in the WT (Figure 4F).

### Overexpression of *CaALAD* Is Involved in the Regulation of Antioxidative Systems in Transgenic *Arabidopsis* Plants

**FIGURE 5:**
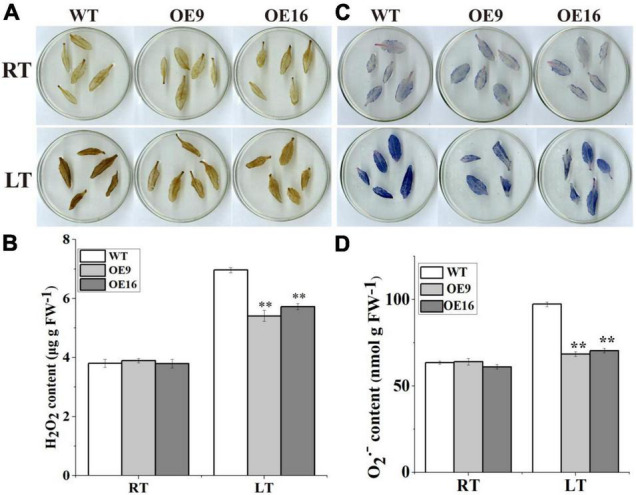
The contents of ROS and the activities of antioxidant enzymes including in the wild-type (WT) and transgenic plants under 4*^o^*C for 1 day. **(A)** DAB staining; **(B)** the content of H_2_O_2_; **(C)** NBT staining; and **(D)** the content of O_2_^–^. The error bars represent the standard errors for three independent tests, and the asterisks (^**^) indicate significant differences from the WT at *p* < 0.01.

**FIGURE 6:**
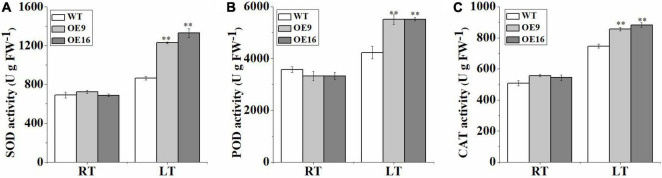
The activities of the antioxidant enzymes SOD **(A)**, POD **(B)**, and CAT **(C)** in the wild-type (WT) and transgenic plants exposed to 4°C for 1 day. The error bars represent the standard errors of three independent tests and the asterisks (^**^) indicate significant differences from the WT at *p* < 0.01.

To further determine whether *CaALAD* is involved in regulating cold tolerance in *Arabidopsis*, we performed histochemical staining and determined the contents of H_2_O_2_ and O_2_^•–^ and the activities of the antioxidant enzymes SOD, POD, and CAT after 24 h of low temperature stress. The transgenic (OE) plants stained less intensely with both NBT and DAB compared to the WT plants following exposure to low temperature stress (Figures 5A,B). The contents of H_2_O_2_ and O_2_^•–^ and the activities of SOD, POD, and CAT were not significantly different between WT and transgenic lines in the control room temperature treatment (Figures 5, 6). The contents of H_2_O_2_ and O_2_^•–^ and the activities of SOD, POD, and CAT increased in both WT and transgenic plants after 24 h of low temperature treatment (Figures 5, 6). The H_2_O_2_ and O_2_^•–^ contents in WT were significantly higher than in the transgenic plants, and the activities of SOD, POD, and CAT were significantly lower than in the transgenic plants after 1 day at 4*^o^*C (Figures 5, 6). After low temperature stress, the O_2_^•–^ content of the OE9 and OE16 plants decreased by 29.6 and 27.5%, respectively, compared with WT, and the H_2_O_2_ content decreased by 22.3 and 17.8%, respectively, compared with WT (Figures 5B,D). In addition, after 24 h at 4*^o^*C, the activities of antioxidant enzymes in OE9 and OE16 plants showed increases of 42.4 and 54.0% (SOD), 30.3 and 30.5% (POD), and 14.6 and 18.3% (CAT), respectively, compared with WT plants (Figure 6).

### Overexpression of *CaALAD* Is Involved in the Regulation of Chlorophyll Biosynthesis in Transgenic *Arabidopsis* Plants

**FIGURE 7:**
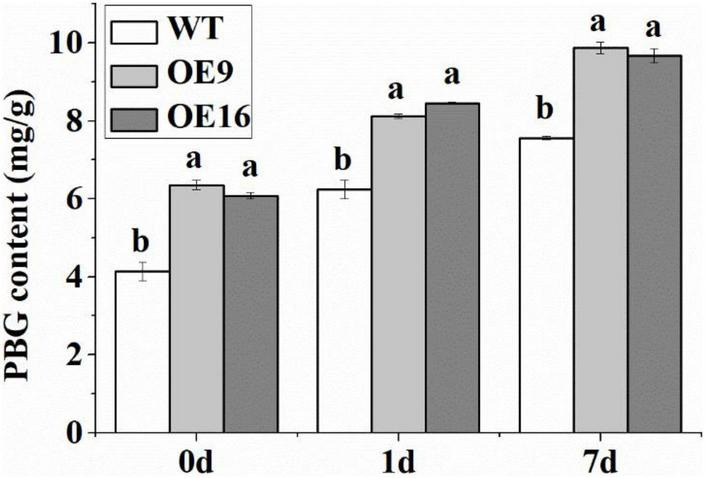
The PBG content of the wild-type (WT) and transgenic plants in response to 4*^o^*C treatment for 0, 1, and 7 day. OE: the *CaALAD*-overexpressing (OE) lines. The error bars represent the standard errors for three independent tests. Different letters show significant differences (*p* < 0.05) based on Duncan’s test.

**FIGURE 8:**
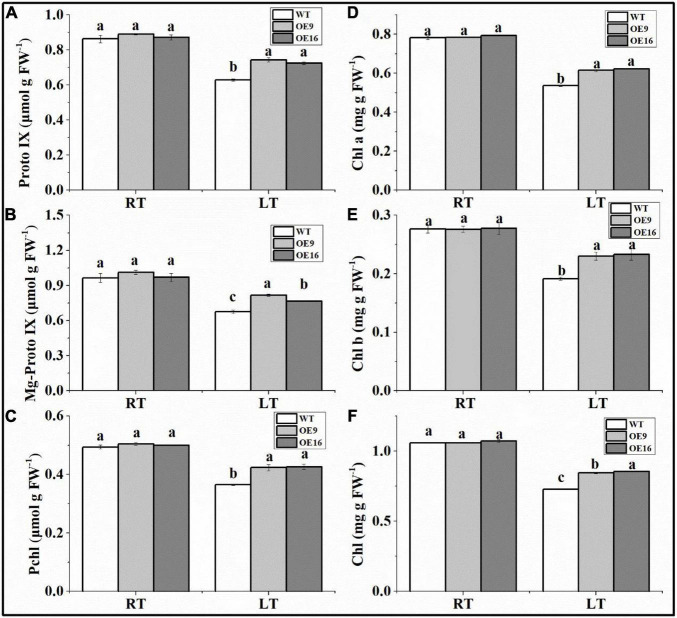
The contents of Proto IX **(A)**, Mg-proto IX **(B)**, Pchl **(C)**, Chl a **(D)**, Chl b **(E)**, and total Chl **(F)** in the wild-type (WT) and transgenic plants exposed to 4°C for 7 day (LT). OE: the *CaALAD*-overexpressing (OE) lines. The error bars represent the standard errors for three independent tests. Different letters show significant differences (*p* < 0.05) based on Duncan’s test.

The ALAD protein catalyzes the condensation of two molecules of ALA into PBG, which can affect the biosynthesis of chlorophyll. In this study, the contents of PBG, Proto IX, Mg-proto IX, Pchl, and Chl in transgenic and WT *Arabidopsis* plants subjected to low temperature stress were measured. The results showed that the PBG content of the transgenic plants was significantly higher than in WT plants under both normal and low temperature conditions (Figure 7). The PBG contents of WT and transgenic *Arabidopsis* plants increased significantly after 1 day of low temperature treatment (Figure 7). In addition, the contents of Proto IX, Mg-proto IX, Pchl, and Chl in the transgenic plants under low temperature stress were significantly higher than in WT plants (Figure 8). After 7 days of low temperature stress, Proto IX in the OE9 and OE16 transgenic plants increased by 18.1 and 15.3%, Mg-proto IX increased by 20.9 and 13.2%, Pchl increased by 15 and 16.8%, Chl a increased by 14.6 and 16.1%, Chl b increased by 20.1 and 21.6%, and total Chl increased by 16 and 17.5%, respectively, compared to WT (Figure 8).

### The Response of Seeds From *CaALAD*-Overexpressing Plants to Exogenous ALA and NaHS Treatments

**FIGURE 9:**
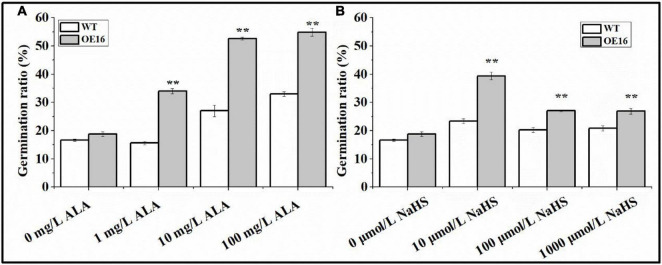
The response of seeds from *CaALAD*-overexpressing plants to exogenous ALA and NaHS. **(A)** The germination rates of transgenic seeds treated with 1 mg/L ALA, 10 mg/L ALA, and 100 mg/L ALA. **(B)** The germination rates of transgenic seeds treated with 10 μmol/L NaHS, 100 μmol/L NaHS, and 1,000 μmol/L NaHS. WT: *Arabidopsis* Col-0. OE: the *CaALAD*-overexpressing (OE) lines. NaHS: the donor of hydrogen sulfide. The error bars are the standard errors for three independent tests and asterisks indicate significant differences from the WT at **p < 0.01.

Wild-type and transgenic *Arabidopsis* seeds were treated with exogenous ALA and NaHS to determine their sensitivity to ALA and H_2_S. The results of this experiment suggest that exogenous ALA and NaHS treatment can significantly improve the germination rate of *Arabidopsis* seeds, while the germination rate of the transgenic seeds was always higher than that of WT seeds (Figure 9). The germination rate of *Arabidopsis* seeds treated with exogenous ALA was the highest at 100 mg/L (Figure 9A). The germination rates of transgenic seeds treated with ALA at 1, 10, and 100 mg/L increased by 117.3, 94.7, and 66.5% compared with WT seeds, respectively, (Figure 9A). The germination rate of transgenic seeds treated with 10 μmol/L NaHS was the highest (Figure 9B). The germination rates of transgenic seeds treated with 10 μmol/L NaHS, 100 μmol/L NaHS, and 1,000 μmol/L NaHS increased by 68.7, 33.6, and 29.3% compared with WT seeds, respectively, (Figure 9B).

### Overexpression of *CaALAD* Regulates the Hydrogen Sulfide Content in Transgenic *Arabidopsis* Under Cold Stress

**FIGURE 10:**
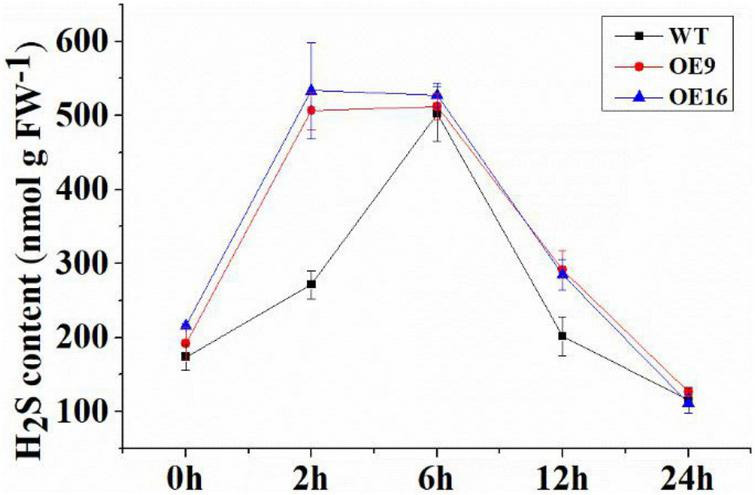
The H_2_S contents of the wild-type (WT) and transgenic plants on cold stress for 0, 2, 6, 12, and 24 h. OE: the *CaALAD*-overexpressing (OE) lines. The error bars are the standard errors for three independent tests.

During low temperature treatment at 4*^o^*C, the H_2_S contents of WT and transgenic plants increased rapidly at 2 h, then decreased sharply at 6 h, and reached a low level at 24 h (Figure 10). However, compared with WT, the H_2_S contents of the transgenic lines were ∼2-fold higher at 2 h than in the WT: the levels in OE9 and OE16 plant increased by 86.7 and 96.6%, respectively, (Figure 10). The H_2_S content of WT plants then increased to the same level as that of the transgenic lines at 6 h, while the H_2_S contents of the transgenic OE plants were the same at 2 h and 6 h (Figure 10).

## Discussion

The ALAD protein plays an important role in higher organisms, and the enzymatic activity of ALAD determines the metabolism of ALA in plants, thus affecting the synthesis of tetrapyrrole molecules, especially chlorophyll (Long et al., 2013). Studies have shown that ALA plays an important role in alleviating abiotic stress, including low temperature stress (Wu et al., 2018b). ALAD can catalyze the condensation of ALA into PBG, which is likely to be involved in the plant response to low temperature stress. Our previous study found that ALAD may be induced by ALA and H_2_S to alleviate the low temperature stress of pepper seedlings (Wang et al., 2021b). In our study, we found that transgenic plants overexpressing *CaALAD* grew better under low temperature stress, with more and longer roots, indicating that the *CaALAD* protein alleviates the inhibition of low temperature on the growth of *Arabidopsis* seedlings (Figure 3). By analyzing the amino acid sequence of CaALAD and comparing it with homologous proteins from other plant species, we found that *ALAD* is a conserved gene, which is consistent with the results of Long et al. (2013). In addition, we found that it is most similar to the protein from eggplant, a related species in the *Solanaceae* family. Moreover, the results of subcellular localization experiments and software prediction showed that the CaALAD protein is localized to the chloroplast, which is an organelle that is highly sensitive to low temperature stress. This also provides an indication that CaALAD is involved in the response to chilling stress in pepper.

Abiotic stress causes plants to produce large amounts of ROS, and excessive accumulation of ROS can cause oxidative stress and cell death (Gill and Tuteja, 2010). Over the course of evolution, plants developed an effective scavenging system to remove ROS. Antioxidant enzymes such as SOD, POD, and CAT play an important role in scavenging ROS and enhancing stress resistance in plants (Hu et al., 2012; Wang et al., 2012). In this study, low temperature stress caused increases in the levels of O_2_^•–^ and H_2_O_2_, and the transgenic plants showed reduced accumulation of O_2_^•–^ and H_2_O_2_ compared to WT (Figures 4A,B). In addition, compared with WT, plants of the transgenic *Arabidopsis* lines showed significantly increased activities of SOD, POD, and CAT in response to low temperature stress (Figures 4C–E). These results indicate that CaALAD mitigates oxidative damage in the transgenic *Arabidopsis* plants by regulating antioxidant enzyme activity. Chai et al. (2017) found that mutation of the *ALAD* gene in cotton leads to a large accumulation of ROS, which is consistent with the results of our study, and indicates that the ALAD protein may be involved in maintaining the ROS balance in plant cells (Chai et al., 2017).

Plants exposed to low temperatures can improve their cold tolerance, which involves changes in plant gene expression (Ma et al., 2018). ICE-CBF-COR is the most important cold defense mechanism in plants. *CBF* (C-repeat binding factor) is an important transcription factor in the plant response to low temperature stress and plays a core role in the low temperature stress regulatory network in plants (Lee et al., 2012; Zhou, 2013). When plants are subjected to low temperature stress, CBF transcription factors are rapidly induced and bind to *cis*-acting elements in the *COR* (Cold-regulated) gene promoter, thereby activating the expression of downstream genes and ultimately improving plant cold tolerance (Shi et al., 2018; Chen et al., 2019; Ye et al., 2021). In this study, expression of both *CBF1* and *CBF2* increased rapidly after 2 h of low temperature stress, while the relative increase in the transgenic *Arabidopsis* plants was significantly higher than in WT. *ICE1* and *ICE2* are positive regulators of *CBF*, although they are not directly regulated by low temperature. *COR15* is a chloroplast-localized specific cold resistance gene, which can enhance the cold resistance of chloroplasts and reduce the cold-induced chloroplast membrane damage by changing the location of membrane lipids (Zhou, 2013; Wang et al., 2017). In this study, the expression levels of the cold tolerance genes *AtCBF1*, *AtCBF2*, *AtICE2*, *AtCOR15a*, and *AtCOR15b* increased rapidly in response to low temperature stress (Figure 4). Compared with WT plants, OE transgenic line plants showed increased transcription of *AtCBF2*, *AtICE1*, and *AtCOR15b* at low temperature (Figure 4). This suggests that the CaALAD protein regulates the expression of cold tolerance genes, and thus participates in the ICE-CBF-COR cold defense mechanism.

Low temperature stress will destroy the photosynthetic system of plants and reduce their photosynthetic capacity. The chloroplast is an essential part of photosynthesis and plays a crucial role in the development and growth of plants through net carbon fixation, biosynthesis of fatty acids, and other physiological processes (Jarvis and López-Juez, 2013). Low temperature is an important environmental factor that affects chloroplast development (Liu et al., 2018). Chlorophyll synthesis is severely reduced under low temperature stress (Tang et al., 2021). ALA is a precursor in the chlorophyll biosynthesis pathway. Lu (2013) concluded that ALAD protein enzyme activity and the PBG content of garlic were significantly increased at low temperature (Lu, 2013). Consistent with this result, we also found that the PBG content increased significantly after 24 h of low temperature stress (Figure 6). The studies of Cui et al. (2001) and He et al. (2008) found that chlorophyll content decreased significantly after low temperature stress, but that the content of PBG, a precursor of chlorophyll synthesis, increased significantly, which was also consistent with the results of our study (Cui et al., 2001; He et al., 2008). Four PBG molecules combine to form a hydroxymethylbilane (HMB), which goes through three steps to form Proto IX (Kobayashi and Masuda, 2016). Mg^2+^ is inserted into Proto IX to form Mg-proto IX, which forms prochloroimide (Pchl), the key precursor of mature Chl, through a three-step reaction (Wu et al., 2018b). Chlorophyll is eventually formed by the activity of chlorophyll synthase. In this study, low temperature (4*^o^*C) significantly reduced the levels of Proto IX, Mg-proto IX, Pchl, and chlorophyll, while the damage to chlorophyll synthesis caused by low temperature was alleviated in *CaALAD*-OE transgenic plants. Chai et al. (2017) found that mutations in *ALAD* reduced chlorophyll content and caused necrotic spots on leaves in cotton, suggesting that *ALAD* plays an important role in maintaining chlorophyll synthesis (Chai et al., 2017).

Zhao et al. (2013) found that treatment with ALA can improve the germination index of tomato seeds (Zhao et al., 2013). Treating seeds with hydrogen sulfide improved the germination rates in rice and maize by regulating the antioxidant system and changing physicochemical properties during seed germination (Liu, 2017; Wang, 2018). Our results also showed that both ALA and H_2_S could significantly improve seed germination rate in *Arabidopsis*. Our previous study showed that exogenous application of ALA and NaHS increased the transcription level of *ALAD* (*HEMB*) in pepper seedlings under low temperature stress (Wang et al., 2021b). Therefore, transgenic and WT seeds were treated with exogenous ALA and NaHS. The effects of ALA and NaHS on the germination rate were quantified to determine whether *CaALAD* responds to ALA and NaHS. The results showed that the transgenic seeds showed a higher germination rate. Due to its gaseous properties, H_2_S can shuttle to all parts of plant cells and is an important signal molecule involved in plant growth and development and the response to stress (Pandey and Gautam, 2020; Zhang et al., 2021). In this study, the content of H_2_S increased rapidly after 2 h of low temperature stress, which is consistent with the results of previous studies; that is, exposure to low temperatures can rapidly induce a transient rise in H_2_S levels (Fu et al., 2013; Du et al., 2017; Wang et al., 2021b). Moreover, we found that the H_2_S content of the transgenic plants changed more rapidly than it did in WT plants at low temperature, suggesting that H_2_S may be involved with the ALAD protein as an important signaling molecule to regulate the plant response to low temperature stress.

## Conclusion

Our study is the first to show that expression of the *ALAD* gene responds to low temperature stress. *ALAD* improves cold tolerance by participating in the regulation of the transcription of cold tolerance genes, enhancing antioxidant systems, and enhancing chlorophyll synthesis. In addition, *ALAD* may also respond to low temperature stress by participating in a hydrogen sulfide-mediated signaling pathway. These results suggest that *ALAD* is an important gene for the response to low temperature stress in pepper.

## Data Availability Statement

The original contributions presented in the study are included in the article/Supplementary Material; further inquiries can be directed to the corresponding author.

## Author Contributions

HW, ZL, and JX designed the research. HW and ZL performed the data analysis. HW wrote the manuscript. JL, JZ, JY, LH, and GZ revised the manuscript. All authors have read and agreed to this version of the manuscript.

## Conflict of Interest

The authors declare that the research was conducted in the absence of any commercial or financial relationships that could be construed as a potential conflict of interest.

## Publisher’s Note

All claims expressed in this article are solely those of the authors and do not necessarily represent those of their affiliated organizations, or those of the publisher, the editors and the reviewers. Any product that may be evaluated in this article, or claim that may be made by its manufacturer, is not guaranteed or endorsed by the publisher.

## Footnotes

1
https://www.arabidopsis.org/

2
https://www.ncbi.nlm.nih.gov/Structure/cdd/wrpsb.cgi

3
https://swissmodel.expasy.org/
